# Long Non-coding RNA ST8SIA6-AS1 Promotes Lung Adenocarcinoma Progression Through Sponging miR-125a-3p

**DOI:** 10.3389/fgene.2020.597795

**Published:** 2020-12-08

**Authors:** Qifeng Cao, Weiqin Yang, Xili Ji, Wei Wang

**Affiliations:** ^1^Department of Respiratory Medicine, Taizhou Hospital of Integrated Traditional Chinese and Western Medicine, Wenling, China; ^2^Department of Gastroenterology, Taizhou Hospital of Integrated Traditional Chinese and Western Medicine, Wenling, China; ^3^Department of Pulmonary Disease, Jinan Traditional Chinese Medicine Hospital, Jinan, China; ^4^Cheeloo College of Medicine, Shandong University, Jinan, China

**Keywords:** long non-coding RNA, lung adenocarcinoma, transcriptional regulation, biomarker, p53

## Abstract

Emerging evidence suggests that long non-coding RNA (lncRNA) plays a critical role in human disease progression. Recently, a novel lncRNA ST8SIA6-AS1 was shown as an important driver in various cancer types. Nevertheless, its contribution to lung adenocarcinoma (LUAD) remains undocumented. Herein, we found that ST8SIA6-AS1 was frequently overexpressed in LUAD cell lines, tissues, and plasma. Depletion of ST8SIA6-AS1 significantly inhibited LUAD cell proliferation and invasion *in vitro* and tumor growth *in vivo*. In term of mechanism, ST8SIA6-AS1 was transcriptionally repressed by tumor suppressor p53, and ST8SIA6-AS1 was mainly located in the cytoplasm and could abundantly sponge miR-125a-3p to increase nicotinamide N-methyltransferase (NNMT) expression, thereby facilitating LUAD malignant progression. Clinically, high ST8SIA6-AS1 was positively correlated with larger tumor size, lymph node metastasis, and later TNM stage. Moreover, ST8SIA6-AS1 was identified as an excellent indicator for MM diagnosis and prognosis. Collectively, our data demonstrate that ST8SIA6-AS1 is a carcinogenic lncRNA in LUAD, and targeting the axis of ST8SIA6-AS1/miR-125a-3p/NNMT may be a promising treatment for LUAD patients.

## Introduction

Lung adenocarcinoma (LUAD) is a type of malignant tumor derived from the bronchial mucosa glandular epithelium, accounting for about 45% of all lung cancers (Coudray et al., 2018). The main treatment methods currently include surgery, radiotherapy, chemotherapy, targeted therapy, and immunotherapy (Zhang et al., 2020a). Despite recent progress in the diagnosis and treatment of LUAD, there has been no significant improvement in survival rate (Bray et al., 2018). Thus, it is of great significance to explore more effective treatment strategies through exploring the potential molecular mechanisms involved in LUAD progression.

Long non-coding RNA (lncRNA) is a non-coding RNA with a length of more than 200 nucleotides (Khorkova et al., 2015). LncRNA sequence is poorly conservative, and only about 12% of lncRNA can be found in organisms other than humans (Jarroux et al., 2017). Most lncRNAs have obvious spatiotemporal expression specificity during tissue differentiation and development (Quinn and Chang, 2016). Recent studies have shown that lncRNA is involved in many important regulatory processes such as chromosome silencing, genome imprinting, chromatin modification, transcription activation/interference, and nuclear transport (Bhan et al., 2017; Qin et al., 2020). The mechanism of action of lncRNA is extremely complicated and has not yet been fully understood, among which the well-verified one is that lncRNA can act as a miRNA molecular sponge and regulate the expression of miRNA targets (Thomson and Dinger, 2016). For example, lnc-GCMA activated by SP1 competitively sponged miR-124 and miR-34a to increase slug and snail expression, thus promoting gastric cancer metastasis *in vitro* and *in vivo* (Tian et al., 2020). LINC00173.v1 increased VEGFA level via sponging miR-511-5p, facilitating angiogenesis and progression of lung squamous cell carcinoma (Chen et al., 2020). Lnc-HSD17B11-1 was significantly upregulated in colorectal cancer and promoted tumorigenesis by absorbing miR-338-3p and increasing MACC1 expression (Zhang et al., 2020b).

Recently, a novel lncRNA, ST8SIA6-AS1, was proposed as an oncogene in multiple human cancers, and depletion of ST8SIA6-AS1 caused mitotic catastrophe, massive apoptosis, and cell cycle arrest (Luo et al., 2020). Up to date, its role in LUAD is still not clear. In this study, we explored the clinical implication and biological effect of ST8SIA6-AS1 in LUAD. We found that it is also a carcinogenic lncRNA in LUAD and further unveiled the underlying mechanism of the pro-tumor effect of ST8SIA6-AS1.

## Materials and Methods

### LUAD Sample

A total of 92 patients with LUAD who underwent resection in Taizhou Hospital of Integrated Traditional Chinese and Western Medicine were selected as the study objects. All cases were confirmed by histopathological diagnosis. The specimens were taken directly from the resected objects, and the normal tissues adjacent to the cancer were ≥ 5 cm away from the lesion, and no tumor cell infiltration was confirmed by pathology. Samples were collected within 10 min after surgical resection and placed in a frozen storage tube, which was stored at -80°C for later use. In addition, plasma samples from LUAD patients and healthy controls were collected to assess the diagnostic value of ST8SIA6-AS1. This study has been approved by the Ethics Committee of Taizhou Hospital of Integrated Traditional Chinese and Western Medicine, and all study participants signed informed consent and were followed up.

### Cell Culture and Transfection

Lung adenocarcinoma cell lines (NCI-H23, HCC827, SPC-A1, and A549) and human bronchial epithelial cells (HBEpC) were all obtained from ATCC. They were cultured in RPMI 1640 culture medium containing 10% fetal bovine serum in an incubator at 37°C and 5% CO_2_. The oligonucleotides including siRNAs (si-SOX2: 5′-GGAGCACCCGGAUUAUAAA-3′; si-Myc: 5′-CAUGGUGAACCAGAGUUUC-3′; si-p53: 5′-UCU ACAAGCAGUCACAGCA-3′; si-STAT3: 5′-GGAGGCAUUC GGAAAGUAU-3′), ASO-ST8SIA6-AS1 (5′-GGGUUUGUG CAAGCAAACU-3′), and miR-125a-3p mimics/inhibitors were designed and commercially purchased from RiboBio (Guangzhou, China). p53-overexpressing pcDNA 3.0 vector was obtained from Thermo Fisher (MA, United States). Transfection was performed using Lipofectamine 3000 (Thermo Fisher) with 10 nM final concentration as per standard protocol, and the transfection efficiency was detected after 48 h using real-time quantitative PCR (qRT-PCR) analysis.

### qRT-PCR Analysis

Total RNA was extracted by TRIzol solution (Invitrogen, CA, United States). The cDNA was then synthesized using Transcriptor First Strand cDNA Synthesis Kit (Roche, Basel, Switzerland) according to the supplier’s protocol, followed by quantification using SYBR Green I Master Mix on a LightCycler 480 (Roche) with 40 cycles (95°C 15 s, 60°C 30 s, and 72°C 30 s). GAPDH and U6 were used as the internal references for lncRNA/mRNA and miRNA, respectively. The relative gene expression was calculated by 2^–ΔΔCT^ method.

### Determination of the Location of ST8SIA6-AS1

Cytoplasmic and nuclear RNA fractions were separated according to the instructions of the PARIS^TM^ Kit (Invitrogen), followed by qRT-PCR analysis. U1 RNA was used as the nuclear endogenous control. GAPDH mRNA was used as the cytoplasmic endogenous control.

### Luciferase Reporter Assay

The two predicted p53 binding sites on ST8SIA6-AS1 promoter were respectively mutated and then cloned into pGL3-basic vector (Promega, WI, United States), followed by co-transfection with p53-overexpressing vector into LUAD cells using Lipofectamine 3000 (Thermo Fisher). For assessing the correlation between ST8SIA6-AS1 and miR-125a-3p, the full-length of ST8SIA6-AS1 with wild-type or mutant miR-125a-3p binding site was embedded into pmirGLO vector (Promega), and then, co-transfection with miR-125a-3p mimics into LUAD cells. After 48 h of transfection, the luciferase activity was tested by Luciferase Reporter Assay Kit (GeneCopoeia, MD, United States).

### Chromatin Immunoprecipitation Assay

The chromatin immunoprecipitation (ChIP) assay was conducted by using SimpleChIP^®^ Plus Enzymatic Chromatin IP Kit with agarose beads (Cell Signaling Technology, MA, United States) following the supplier’s protocol with minor modification. In brief, LUAD cell lysates were treated with 1% formaldehyde and glycine, followed by chromatin digestion using micrococcal nuclease. Afterward, 5 μg p53 antibody (sc-126, TX, United States) was added into cell lysates and incubated overnight. After washing, the DNA fragment pulled down by p53 antibody was eluted for PCR analysis.

### Cell Proliferation

For CCK-8 assay, LUAD cells were digested by trypsin; then, 100-μl cell suspension was seeded into 96-well plates and cultured for 24, 48, and 72 h. After that, 10-μl CCK-8 solution was added and incubated for 0.5–4 h depending on the color change. The 96-well plates were taken out from cell incubator and put into a microplate reader for detection of optical density value. For colony formation assay, 500 cells were plated onto six-well plates and cultured for 2 weeks, followed by staining with crystal violet. The colony number was counted in each plate.

### Transwell Assay

The transwell chamber coated with polycarbonate membrane was put into the 24-well plates. The upper chamber was added with 200 μl serum-free cell suspension, and the lower chamber was added with 600 μl complete medium. Then, the 24-well plates were plated into cell incubator and cultured for 20 h. The non-migrated cells were erased and the migrated cells were stained by crystal violet.

### RNA Pull-Down Assay

The biotin-labeled probe against ST8SIA6-AS1 was synthesized (RiboBio, Guangzhou, China) and incubated with NCI-H23 and A549 cell lysates overnight with rotation. After that, cell lysates were added with streptavidin-coupled Dynabeads (Invitrogen), followed by incubation for 3 h. The miRNAs pulled down by ST8SIA6-AS1 probe were isolated by TRIzol reagent, and qRT-PCR analysis was carried out.

### Western Blot

Total protein was extracted by RIPA reagent and the concentration was detected by BCA method as per supplier’s protocol (Beyotime, Beijing, China). For electrophoresis, 20-μg protein was added into each lane, followed by transfer onto polyvinylidene fluoride (PVDF) membrane. After sealing by 5% skim milk powder, the PVDF membrane was incubated with nicotinamide N-methyltransferase (NNMT) antibody (ab119758, Abcam) overnight at 4°C. After TBST washing, the PVDF membrane was incubated with secondary antibody for 1 h. The signal was visualized by enhanced chemiluminescence (ECL) reagent (Pierce, MA, United States).

### Immunohistochemistry

Lung adenocarcinoma tissue specimens were routinely embedded in paraffin and sectioned with a thickness of 4 μM. Then, the pathological section was incubated with NNMT antibody (ab119758, Abcam), and PBS was used instead of the primary antibody as negative control. The signal was visualized by DAB dye. The immunohistochemistry (IHC) results were analyzed by two senior pathologists under the double-blind condition.

### Xenograft Tumor Model

A total of six nude mice were purchased from the Animal Model of the Chinese Academy of Sciences (Shanghai, China), and they were randomly divided into two groups (*n* = 3 per group). NCI-H23 cells were subcutaneously injected into nude mice; after 1 week, intratumoral injection of ASO-control and ASO-ST8SIA6-AS1 was respectively conducted. After 5 weeks, mice were euthanized, and tumor tissues were collected for qRT-PCR and western blot analysis.

### Bioinformatics Analysis

To analyze which transcription factors were involved in the regulation of ST8SIA6-AS1, three online tools including PROMO^1^, JASPAR^2^, and TRANSFAC^3^ were used by inputting the promoter sequence of ST8SIA6-AS1. To evaluate the downstream miRNAs of ST8SIA6-AS1, three online tools including RegRNA^4^, TargetScan^5^, and LncRNASNP^6^ were used by inputting the full length of ST8SIA6-AS1. The predicted results were generated automatically, followed by intersection.

### Statistical Analysis

All data were analyzed by SPSS17.0 software. All results are the mean ± SD of at least three independent experiments carried out in triplicate. The comparison between the two groups was performed by Student’s *t*-test. The correlations between ST8SIA6-AS1 and the clinicopathological data was analyzed by chi-square test. *P* < 0.05 indicated that the difference was statistically significant.

## Results

### ST8SIA6-AS1 Is Overexpressed in LUAD Tissues, Cells, and Plasma

**FIGURE 1:**
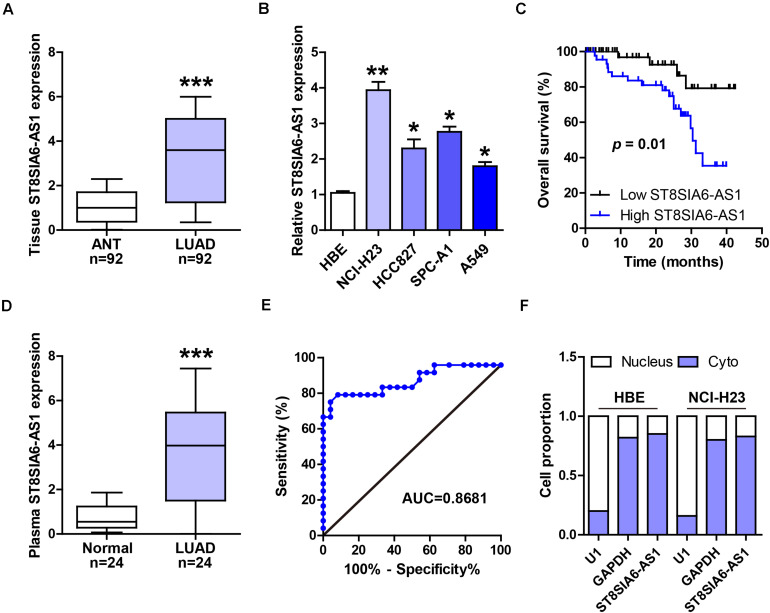
ST8SIA6-AS1 is overexpressed in LUAD. **(A,B)** qRT-PCR analysis of ST8SIA6-AS1 expression in LUAD tissues and cell lines. **(C)** The survival curve of LUAD patients with low and high ST8SIA6-AS1 level. **(D)** qRT-PCR analysis of ST8SIA6-AS1 expression in LUAD plasma. **(E)** ROC curve analyzing the diagnostic value of plasma ST8SIA6-AS1 for LUAD. **(F)** qRT-PCR analysis testing the location of ST8SIA6-AS1 in HBE and NCI-H23 cells. *P < 0.05, **P < 0.01, ***P < 0.001.

A total of 92 matched LUAD and normal tissues were collected for qRT-PCR analysis of ST8SIA6-AS1 level, and high ST8SIA6-AS1 expression was observed in LUAD tissues as compared to normal tissues (Figure 1A). Similarly, ST8SIA6-AS1 was uniformly upregulated in different LUAD cell lines (Figure 1B). Patients with high ST8SIA6-AS1 level were more likely to develop larger tumor sizes, later clinical stage, and lymph node metastasis (Table 1), and ST8SIA6-AS1 expression was positively correlated with dismal prognosis (Figure 1C). To test whether ST8SIA6-AS1 could be used as a non-invasive marker, we collected plasma samples from LUAD patients and healthy control and conducted qRT-PCR assay. The results showed that ST8SIA6-AS1 was also increased in LUAD plasma (Figure 1D); the area under curve (AUC) was 0.8681 (95% CI: 0.7566 to 0.9796) (Figure 1E), suggesting that plasma ST8SIA6-AS1 is an excellent diagnostic indicator. Besides, we detected the location of ST8SIA6-AS1 in normal and LUAD cells; the results showed that ST8SIA6-AS1 is a cytoplasmic lncRNA (Figure 1F).

**TABLE 1:** The correlations between ST8SIA6-AS1 expression and clinicopathological features in LUAD patients (*n* = 92).

Parameters	All cases (*n* = 92)	ST8SIA6-AS1 expression		*P-*value
		Low (*n* = 46)	High (*n* = 46)	
**Gender**				
Male	57	26	31	0.283
Female	35	20	15	
**Age (years)**				
≤ 65	53	27	26	0.833
> 65	39	19	20	
**Tumor size**				
≤ 3	44	28	16	0.012
> 3	48	18	30	
**Lymph node metastasis**				
No	52	33	19	0.003
Yes	40	13	27	
**TNM stage**				
I-II	54	33	21	0.011
III-IV	38	13	25	

### ST8SIA6-AS1 Is Transcriptionally Inhibited by p53

**FIGURE 2:**
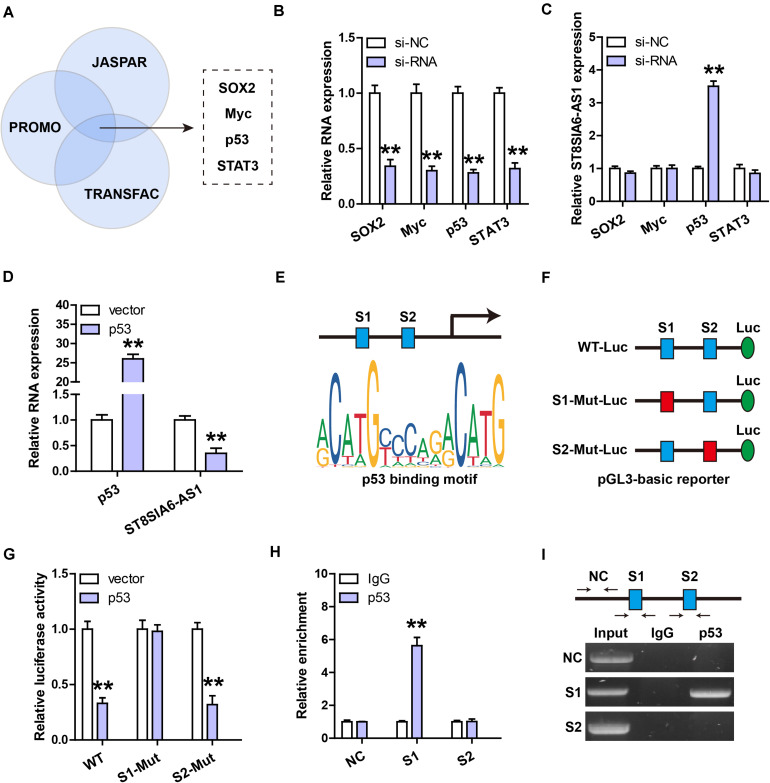
ST8SIA6-AS1 is inhibited by p53. **(A,B)** Three databases predicting the transcription factors targeting ST8SIA6-AS1. **(B)** qRT-PCR analysis verifying the silencing efficiency of these indicated four siRNAs. **(C)** qRT-PCR analysis of ST8SIA6-AS1 expression after knockdown of SOX2, Myc, p53, or STAT3. **(D)** qRT-PCR analysis of ST8SIA6-AS1 expression after p53 overexpression. **(E)** Two predicted p53 binding motifs on ST8SIA6-AS1 promoter. **(F,G)** Luciferase reporter assay was performed using the indicated wily-type and mutant pGL3-basic vector. **(H,I)** ChIP assay using p53 antibody, followed by PCR analysis of the enrichment of p53 on ST8SIA6-AS1 promoter. **P < 0.01.

Through analyzing three online databases of transcriptional regulation, we found that ST8SIA6-AS1 might be modulated by four transcription factors, including SOX2, Myc, p53, and STAT3 (Figure 2A). We then performed siRNA screening (Figure 2B), and the results showed that only silencing of p53 could significantly affect ST8SIA6-AS1 level (Figure 2C). Consistently, ST8SIA6-AS1 was substantially decreased after exogenous p53 overexpression (Figure 2D). There are two p53 binding sites on ST8SIA6-AS1 promoter (Figure 2E); we mutated them individually and performed luciferase reporter assay (Figure 2F). The results displayed that overexpression of p53 significantly decreased the luciferase activity of the wild-type reporter, whereas this effect disappeared after site 1 mutation, but not after site 2 mutation (Figure 2G). Further, the ChIP-PCR assay showed that p53 was abundantly enriched on site 1, not site 2 (Figures 2H,I).

### ST8SIA6-AS1 Promotes LUAD Cell Proliferation and Invasion

**FIGURE 3:**
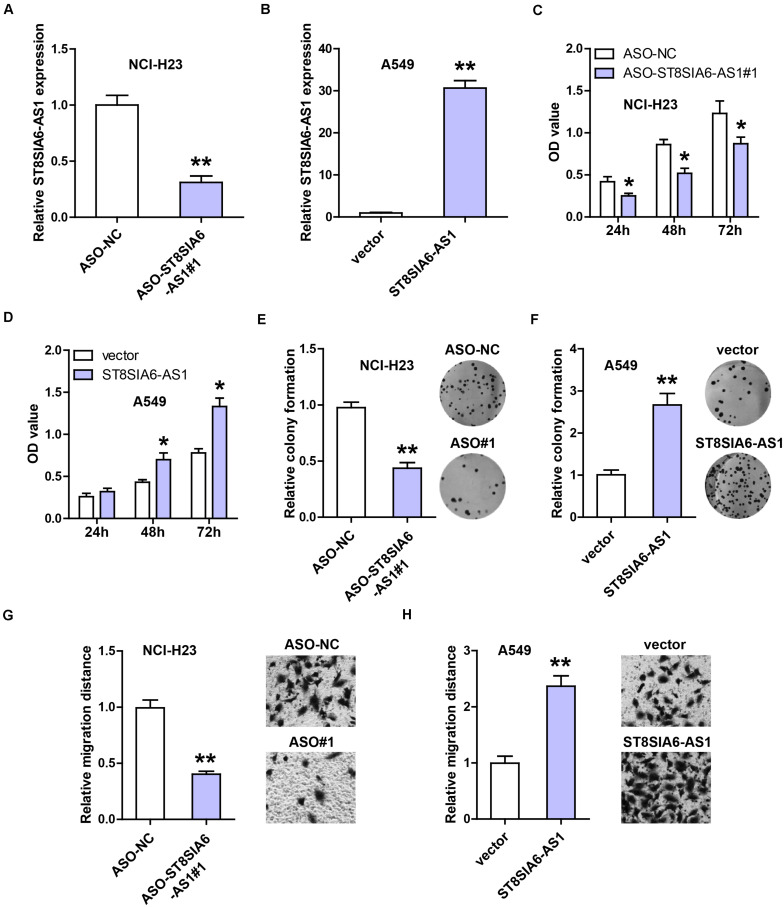
ST8SIA6-AS1 promotes LUAD cell malignant phenotype. **(A,B)** qRT-PCR analysis verifying the silencing and overexpression efficiencies in NCI-H23 and A549 cells, respectively. **(C–H)** CCK-8, colony formation, and transwell assays testing cell viability, colony ability, and migration after alteration of ST8SIA6-AS1 level, respectively. *P < 0.05, **P < 0.01.

Then, we chose NCI-H23 and A549 cells to silence and overexpress ST8SIA6-AS1, respectively (Figures 3A,B). The CCK-8 assay was carried out and the results showed that knockdown of ST8SIA6-AS1 significantly reduced NCI-H23 cell viability (Figure 3C), whereas enforced expression of ST8SIA6-AS1 increased A549 cell viability (Figure 3D). Similarly, LUAD cell colony ability was weakened after ST8SIA6-AS1 silencing (Figure 3E) but was enhanced after ST8SIA6-AS1 overexpression (Figure 3F). In addition, we also detected cell migration after alteration of ST8SIA6-AS1 expression; as shown in Figure 3G, the migration distance was significantly shortened in ST8SIA6-AS1-depleted cells as compared to control cells. Likewise, the exogenous expression of ST8SIA6-AS1 resulted in an opposite trend (Figure 3H).

### ST8SIA6-AS1 Acts as a Sponge of miR-125a-3p

**FIGURE 4:**
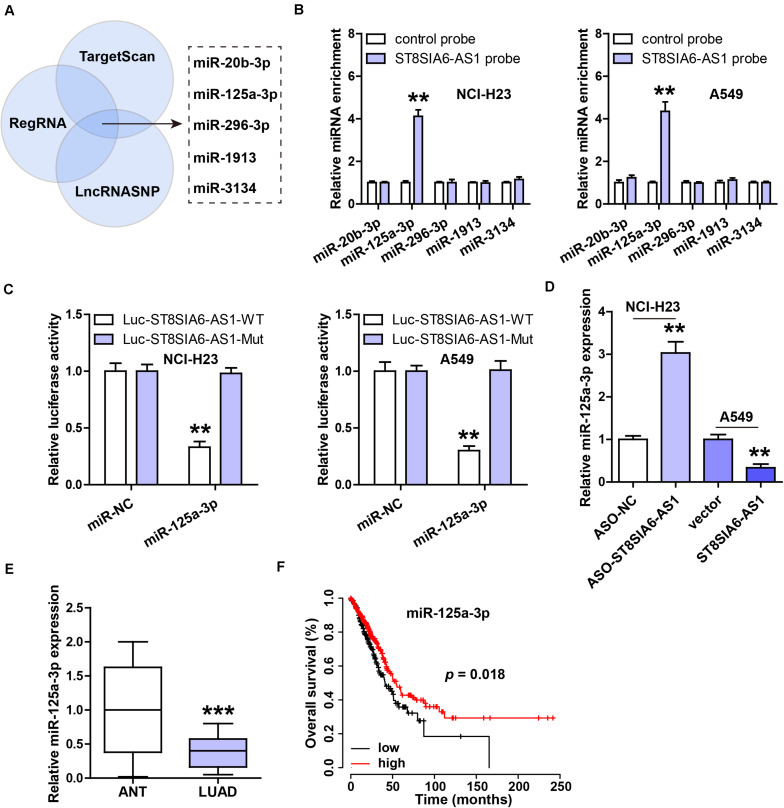
ST8SIA6-AS1 sponges miR-125a-3p in LUAD cells. **(A)** Three online databases predicting miRNAs bound by ST8SIA6-AS1. **(B)** RNA pull-down assay using biotinylated ST8SIA6-AS1 probe, followed by qRT-PCR analysis. **(C)** Luciferase reporter assay testing the inhibitory effect of miR-125a-3p on ST8SIA6-AS1. **(D)** qRT-PCR analysis of miR-125a-3p level after alteration of ST8SIA6-AS1 expression. **(E)** qRT-PCR analysis of miR-125a-3p expression in LUAD and matched normal tissues. **(F)** The survival curve of LUAD patients based on miR-125a-3p level. **P < 0.01, ***P < 0.001.

In light of the cytoplasmic location of ST8SIA6-AS1, we inferred that ST8SIA6-AS1 might serve as a miRNA sponge. By analyzing three online forecasting tools, we found five miRNAs that might be complementary to ST8SIA6-AS1 (Figure 4A). We then performed RNA pull-down assay, and the results showed that only miR-125a-3p was abundantly enriched by ST8SIA6-AS1 probe in both NCI-H23 and A549 cells (Figure 4B). Next, the binding site of miR-125a-3p on ST8SIA6-AS1 was mutated, followed by luciferase reporter assay. The results displayed that miR-125a-3p overexpression significantly reduced the luciferase activity of the wild-type reporter, but did not affect the mutant one (Figure 4C). Moreover, miR-125a-3p level was notably increased after ST8SIA6-AS1 depletion, but decreased after ST8SIA6-AS1 overexpression (Figure 4D). Besides, we tested the expression of miR-125a-3p in LUAD tissues; as shown in Figure 4E, its expression was dramatically decreased in LUAD compared to matched normal tissues, and LUAD patients with high miR-125a-3p had longer overall survival time than those with low miR-125a-3p (Figure 4F).

### ST8SIA6-AS1 Regulates the miR-125a-3p/NNMT Axis

**FIGURE 5:**
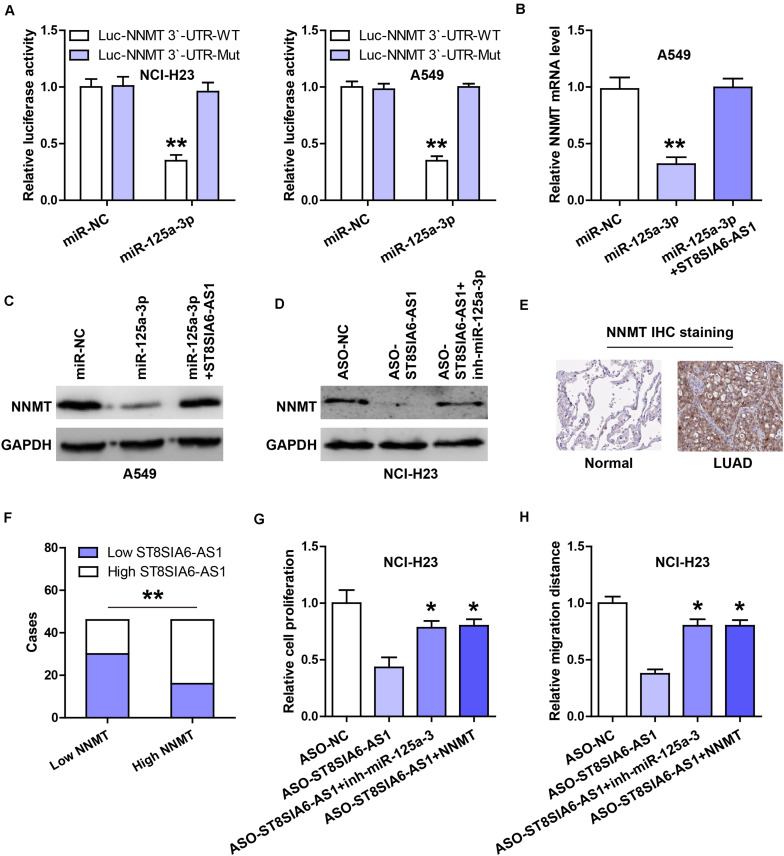
ST8SIA6-AS1 regulates the miR-125a-3p/NNMT axis. **(A)** Luciferase reporter assay testing the inhibitory effect of miR-125a-3p on NNMT 3’-UTR. **(B,C)** qRT-PCR and western blot analysis of NNMT mRNA and protein levels in miR-125a-3p-silenced A549 cells transfected with ST8SIA6-AS1 expression vector, respectively. **(D)** Western blot analysis of NNMT protein level in ST8SIA6-AS1-depleted NCI-H23 cells transfected with miR-125a-3p inhibitors. **(E)** IHC staining of NNMT in LUAD and normal tissues. **(F)** The correlation between ST8SIA6-AS1 and NNMT protein in LUAD tissues. **(G,H)** CCK-8 and transwell assays respectively testing cell viability and migration in ST8SIA6-AS1-depleted NCI-H23 cells transfected with miR-125a-3p inhibitors or NNMT expression vector. *P < 0.05, **P < 0.01.

Through analyzing the miRWalk database, we found that the well-documented oncogene NNMT might be the downstream target of miR-125a-3p. Then, we performed the luciferase reporter assay, and the results showed that the luciferase activity of the wild-type NNMT 3’-UTR reporter was dramatically decreased after miR-125a-3p overexpression, whereas that of the mutant reporter was not changed (Figure 5A). NNMT mRNA and protein levels were significantly decreased by enforced expression of miR-125a-3p (Figures 5B,C), and these effects were completely blocked after ST8SIA6-AS1 overexpression (Figures 5B,C). Moreover, silencing of ST8SIA6-AS1 reduced NNMT protein expression, and knockdown of miR-125a-3p rescued this phenomenon (Figure 5D). The IHC staining results showed that NNMT protein was significantly increased in LUAD tissues in comparison to normal tissues (Figure 5E), and high NNMT was positively correlated with high ST8SIA6-AS1 (Figure 5F). Functionally, the reduced cell viability and migration caused by ST8SIA6-AS1 knockdown were effectively rescued by miR-125a-3p silencing or NNMT overexpression (Figures 5G,H).

### Depletion of ST8SIA6-AS1 Retards Tumor Growth

**FIGURE 6:**
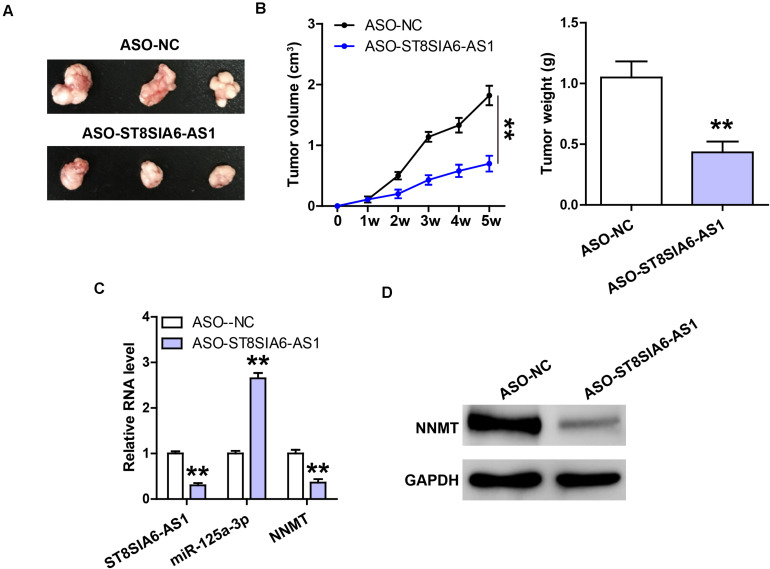
Knockdown of ST8SIA6-AS1 inhibits tumor growth. **(A,B)** Tumor volume and weight of nude mice bearing control or ST8SIA6-AS1-silenced NCI-H23 cells. **(C)** qRT-PCR analysis of ST8SIA6-AS1, miR-125a-3p, and NNMT expression in control or ST8SIA6-AS1-silenced tumor tissues. **(D)** Western blot testing NNMT protein level in control or ST8SIA6-AS1-silenced tumor tissues. **P < 0.01.

Lastly, we established the xenograft tumor model to determine the *in vivo* effect of ST8SIA6-AS1. As shown in Figures 6A,B, the tumor volume and weight of ST8SIA6-AS1-silenced group were significantly smaller than those of the control group. Tumor tissues with low ST8SIA6-AS1 expression had high miR-125a-3p level compared to tissues with high ST8SIA6-AS1 expression (Figure 6C), and NNMT mRNA and protein levels in the ST8SIA6-AS1-depleted group were significantly lower than those in the control group (Figure 6D).

## Discussion

In the present study, we for the first time showed that ST8SIA6-AS1 functioned as an oncogenic lncRNA in LUAD. ST8SIA6-AS1 was reported to be significantly upregulated in various cancer types; however, the mechanisms by which it promoted cancer development and progression were different. In hepatocellular carcinoma, ST8SIA6-AS1 increased HDAC11 expression by targeting miR-4656, thus enhancing hepatocellular carcinoma cell proliferation and resistance to apoptosis (Fei et al., 2020). In colorectal cancer, ST8SIA6-AS1 served as a sponge of miR-5195 and elevated PCBP2, promoting tumor cell proliferation, migration, and invasion (Huang et al., 2020). Another study showed that ST8SIA6-AS1 facilitated breast tumorigenesis via linking up PLK1 and Aurora A to increase PLK1 phosphorylation (Luo et al., 2020). And in our study, we found that ST8SIA6-AS1 could sponge miR-125a-3p, but not miR-4656 or miR-5195, in LUAD cells (data not shown). These indicate that lncRNA functions in a cell- or disease-context-dependent manner (Bhan et al., 2017). Through *in silico* analysis, we found a large number of target genes of miR-125a-3p, among which NNMT, a cytosolic enzyme, most sparked our interest because it has been identified as an oncogene in a series of human cancers, including LUAD (Sartini et al., 2013; Eckert et al., 2019), which is also verified by the results from the UALCAN^7^ online analysis tool (data not shown). Further experiments reveled that NNMT expression was increased by ST8SIA6-AS1 via miR-125a-3p both *in vitro* and *in vivo*, and the attenuated malignant phenotype caused by ST8SIA6-AS1 knockdown was effectively blocked after miR-125a-3p silencing or NNMT overexpression, suggesting that the regulatory axis of ST8SIA6-AS1/miR-125a-3p/NNMT does exist in LUAD. The expression level and potential mechanism of ST8SIA6-AS1 in other malignant tumors need to be further studied.

Nowadays, multiple new cancer-related lncRNAs have been successfully identified by analyzing the data in RNA high-throughput sequencing. Some lncRNAs were found to be abnormally expressed in tumorigenesis and progression, hinting their great potentials as tumor biomarkers (Chandra and Nandan, 2017). For instance, John et al. tested the expression of lnc-SChLAP1 in 1,008 prostate cancer tissues, found that it was notably upregulated, and further identified high SChLAP1 expression as significantly prognostic for metastatic disease progression (Prensner et al., 2014). Besides, HOTAIR, a well-known oncogenic lncRNA, was reported to be frequently overexpressed in various human cancers, linking to poor prognosis, metastasis, and recurrence (Rajagopal et al., 2020). In the current study, we found that ST8SIA6-AS1 was highly expressed in LUAD tissues and cell lines, and patients with high ST8SIA6-AS1 displayed shortened survival time than those with low ST8SIA6-AS1, indicating that it is a promising prognostic biomarker for LUAD. More importantly, high ST8SIA6-AS1 was also found in LUAD plasma, and the AUC value was 0.8681, suggesting that it can be used as a non-invasive indicator for diagnosing LUAD. It is of great interest to test whether ST8SIA6-AS1 is also present and highly expressed in other human body fluids such as urine or sweat. Moreover, multi-center large-scale studies are necessary to verify its potential as an excellent disease biomarker for LUAD.

Gene expression in eukaryotes is strictly regulated mainly at the level of transcription. Likewise, lncRNA level is controlled by some transcription factors, such as p53, a well-recognized tumor suppressor (Zhang et al., 2014). p53 is frequently inactivated or lowly expressed in human cancer, and it is able to directly bind to gene promoters to promote or inhibit gene expression (Vousden and Prives, 2009). Currently, numerous lncRNAs are identified as the targets of p53, such as uc061hsf.1 (Yao et al., 2020), PiHL (Deng et al., 2020), and TRMP (Yang et al., 2018). Herein, two p53 binding motifs on ST8SIA6-AS1 promoter were predicted using *in silico* analysis, and we performed ChIP and luciferase reporter assays and found that p53 could bind to the motif away from the transcription initiation site, inhibiting ST8SIA6-AS1 expression. Therefore, our data suggest that p53 transcriptionally inactivates ST8SIA6-AS1, and the upregulation of ST8SIA6-AS1 in LUAD is attributed to the inactivation of p53.

## Conclusion

In this study, we for the first time uncover the critical tumor-promoting role of ST8SIA6-AS1 in LUAD. Dysregulation of the p53/ST8SIA6-AS1/miR-125a-3p/NNMT signaling axis may contribute to the aggressive progression of LUAD.

## Data Availability Statement

The original contributions presented in the study are included in the article/supplementary material, further inquiries can be directed to the corresponding author/s.

## Ethics Statement

The studies involving human participants were reviewed and approved by the Ethics Committee of Taizhou Hospital of Integrated Traditional Chinese and Western Medicine. The patients/participants provided their written informed consent to participate in this study. The animal study was reviewed and approved by the Ethics Committee of Taizhou Hospital of Integrated Traditional Chinese and Western Medicine.

## Author Contributions

WW and QC designed the research. QC, WY, and XJ performed the assays and analyzed all data. QC drafted this manuscript and WW revised it. All authors contributed to this manuscript and approved the final version.

## Conflict of Interest

The authors declare that the research was conducted in the absence of any commercial or financial relationships that could be construed as a potential conflict of interest.

## Footnotes

1 http://alggen.lsi.upc.es/

2 http://jaspar.genereg.net/

3 http://gene-regulation.com/

4 http://regrna2.mbc.nctu.edu.tw/

5 http://www.targetscan.org/

6 http://bioinfo.life.hust.edu.cn

7 http://ualcan.path.uab.edu/

## References

[B1] Bhan A. Soleimani M. Mandal S. S. (2017). Long noncoding RNA and cancer: a new paradigm. *Cancer Res.* 77 3965–3981. 10.1158/0008-5472.CAN-16-2634 28701486 PMC8330958

[B2] Bray F. Ferlay J. Soerjomataram I. Siegel R. L. Torre L. A. Jemal A. (2018). Global cancer statistics 2018: GLOBOCAN estimates of incidence and mortality worldwide for 36 cancers in 185 countries. *CA Cancer J. Clin.* 68 394–424. 10.3322/caac.21492 30207593

[B3] Chandra G. S. Nandan T. Y. (2017). Potential of long non-coding RNAs in cancer patients: from biomarkers to therapeutic targets. *Int. J. Cancer* 140 1955–1967. 10.1002/ijc.30546 27925173

[B4] Chen J. Liu A. Wang Z. Wang B. Chai X. Lu W. (2020). LINC00173.v1 promotes angiogenesis and progression of lung squamous cell carcinoma by sponging miR-511-5p to regulate VEGFA expression. *Mol. Cancer* 19:98. 10.1186/s12943-020-01217-2 32473645 PMC7260858

[B5] Coudray N. Ocampo P. S. Sakellaropoulos T. Narula N. Snuderl M. Fenyö D. (2018). Classification and mutation prediction from non-small cell lung cancer histopathology images using deep learning. *Nat. Med.* 24 1559–1567. 10.1038/s41591-018-0177-5 30224757 PMC9847512

[B6] Deng X. Li S. Kong F. Ruan H. Xu X. Zhang X. (2020). Long noncoding RNA PiHL regulates p53 protein stability through GRWD1/RPL11/MDM2 axis in colorectal cancer. *Theranostics* 10 265–280. 10.7150/thno.36045 31903119 PMC6929633

[B7] Eckert M. A. Coscia F. Chryplewicz A. Chang J. W. Hernandez K. M. Pan S. (2019). Proteomics reveals NNMT as a master metabolic regulator of cancer-associated fibroblasts. *Nature* 569 723–728. 10.1038/s41586-019-1173-8 31043742 PMC6690743

[B8] Fei Q. Song F. Jiang X. Hong H. Xu X. Jin Z. (2020). LncRNA ST8SIA6-AS1 promotes hepatocellular carcinoma cell proliferation and resistance to apoptosis by targeting miR-4656/HDAC11 axis. *Cancer Cell Int.* 20:232. 10.1186/s12935-020-01325-5 32536820 PMC7288512

[B9] Huang C. M. Cao G. Y. Yang C. X. Chen Y. Liu G. D. Xu B.-W. (2020). LncRNA ST8SIA6-AS1 promotes colorectal cancer cell proliferation, migration and invasion by regulating the miR-5195/PCBP2 axis. *Eur. Rev. Med. Pharmacol. Sci.* 24 4203–4211. 10.26355/eurrev_202004_21000 32373956

[B10] Jarroux J. Morillon A. Pinskaya M. (2017). History, discovery, and classification of lncRNAs. *Adv. Exp. Med. Biol.* 1008 1–46. 10.1007/978-981-10-5203-3_1 28815535

[B11] Khorkova O. Hsiao J. Wahlestedt C. (2015). Basic biology and therapeutic implications of lncRNA. *Adv. Drug Deliv. Rev.* 87 15–24. 10.1016/j.addr.2015.05.012 26024979 PMC4544752

[B12] Luo M. L. Li J. Shen L. Chu J. Guo Q. Liang G. (2020). The role of APAL/ST8SIA6-AS1 lncRNA in PLK1 activation and mitotic catastrophe of tumor cells. *J. Natl. Cancer Inst.* 112 356–368. 10.1093/jnci/djz134 31286138 PMC7156940

[B13] Prensner J. R. Zhao S. Erho N. Schipper M. Iyer M. K. Dhanasekaran S. M. (2014). RNA biomarkers associated with metastatic progression in prostate cancer: a multi-institutional high-throughput analysis of SChLAP1. *Lancet Oncol.* 15 1469–1480. 10.1016/S1470-2045(14)71113-1 25456366 PMC4559342

[B14] Qin T. Li J. Zhang K. Q. (2020). Structure, regulation, and function of linear and circular long Non-Coding RNAs. *Front. Genet.* 11:150. 10.3389/fgene.2020.00150 32194627 PMC7063684

[B15] Quinn J. J. Chang H. Y. (2016). Unique features of long non-coding RNA biogenesis and function. *Nat. Rev. Genet.* 17 47–62. 10.1038/nrg.2015.10 26666209

[B16] Rajagopal T. Talluri S. Akshaya R. L. Dunna N. R. (2020). HOTAIR LncRNA: a novel oncogenic propellant in human cancer. *Clin. Chim. Acta* 503 1–18. 10.1016/j.cca.2019.12.028 31901481

[B17] Sartini D. Morganti S. Guidi E. Rubini C. Zizzi A. Giuliante R. (2013). Nicotinamide N-methyltransferase in non-small cell lung cancer: promising results for targeted anti-cancer therapy. *Cell Biochem. Biophys.* 67 865–873. 10.1007/s12013-013-9574-z 23532607

[B18] Thomson D. W. Dinger M. E. (2016). Endogenous microRNA sponges: evidence and controversy. *Nat. Rev. Genet.* 17 272–283. 10.1038/nrg.2016.20 27040487

[B19] Tian Y. Ma R. Sun Y. Liu H. Zhang H. Sun Y. (2020). SP1-activated long noncoding RNA lncRNA GCMA functions as a competing endogenous RNA to promote tumor metastasis by sponging miR-124 and miR-34a in gastric cancer. *Oncogene* 39 4854–4868. 10.1038/s41388-020-1330-4 32439864

[B20] Vousden K. H. Prives C. (2009). Blinded by the light: the growing complexity of p53. *Cell* 137 413–431. 10.1016/j.cell.2009.04.037 19410540

[B21] Yang Y. Wang C. Zhao K. Zhang G. Wang D. Mei Y. (2018). TRMP, a p53-inducible long noncoding RNA, regulates G1/S cell cycle progression by modulating IRES-dependent p27 translation. *Cell Death Dis.* 9:886. 10.1038/s41419-018-0884-3 30166522 PMC6117267

[B22] Yao J. Zhang H. Li H. Qian R. Liu P. Huang J. (2020). P53-regulated lncRNA uc061hsf.1 inhibits cell proliferation and metastasis in human esophageal squamous cell cancer. *IUBMB Life* 72 401–412. 10.1002/iub.2196 31743955

[B23] Zhang A. Xu M. Mo Y. Y. (2014). Role of the lncRNA-p53 regulatory network in cancer. *J. Mol. Cell Biol.* 6 181–191. 10.1093/jmcb/mju013 24721780 PMC4034727

[B24] Zhang H. Guo L. Chen J. (2020a). Rationale for lung adenocarcinoma prevention and drug development based on molecular biology during carcinogenesis. *Onco Targets Ther.* 13 3085–3091. 10.2147/OTT.S248436 32341654 PMC7166063

[B25] Zhang W. Wang B. Wang Q. Zhang Z. Shen Z. Ye Y. (2020b). Lnc-HSD17B11-1:1 functions as a competing endogenous RNA to promote colorectal cancer progression by sponging miR-338-3p to upregulate MACC1. *Front. Genet.* 11:628. 10.3389/fgene.2020.00628 32595704 PMC7304498

